# Evaluation of the Effectiveness and Color Stability of In-Office Bleaching Agents: A Retrospective Study

**DOI:** 10.3390/jcm15093458

**Published:** 2026-05-01

**Authors:** İdil Gönüllü, Elif Ercan Devrimci, Başak Singün, Ege Türkmen, Hande Kemaloğlu

**Affiliations:** Restorative Department, Ege University, İzmir 35040, Turkey; idil.gonullu@ege.edu.tr (İ.G.); basak.singun@ege.edu.tr (B.S.); ege.turkmen@ege.edu.tr (E.T.); hande.kemaloglu@ege.edu.tr (H.K.)

**Keywords:** tooth bleaching, hydrogen peroxide, tooth discoloration, spectrophotometric analysis, color stability

## Abstract

**Background:** The aim of this study was to evaluate the whitening efficacy and long-term stability of different in-office bleaching agents containing high concentrations of hydrogen peroxide (HP). **Methods:** Records of 50 patients who underwent in-office bleaching treatment at the Department of Restorative Dentistry, Ege University, were retrospectively analyzed. Five bleaching agents were evaluated: Opalescence (40%HP), FGM Whiteness HP (35%HP), FGM Whiteness HP Blue (35%HP), Biowhiten (40%HP) and Powerbright (35%HP). Color measurements were obtained using a spectrophotometer (VITA Easyshade V, VITA Zahnfabrik, Bad Säckingen, Germany) and standardized intraoral photographs at baseline, immediately after treatment, at 1 week, 6 months, 12 months, and 18 months. Color change (ΔE_00_) was calculated using the CIEDE2000 formula, and shade differences were assessed using ΔSGU values. Statistical analyses were performed using repeated-measures ANOVA and Kruskal–Wallis tests, followed by post hoc comparisons where appropriate (*p* < 0.05). **Results:** Baseline age distribution, initial color parameters, and patient-related behavioral factors were comparable among the groups, with no statistically significant differences observed. All bleaching agents resulted in significant color improvement at the 1-week evaluation (ΔE_00_ range: 3.67–6.34; *p* < 0.05), exceeding clinically acceptable thresholds. At 6 months, slight, non-significant reductions in ΔE_00_ values were observed in the Powerbright, FGM Whiteness HP Blue, and FGM Whiteness HP groups (*p* > 0.05). At 18 months, ΔE_00_ values remained within a clinically acceptable range (3.56–4.74), with no significant color regression in most groups (*p* > 0.05), except for a significant decrease in the FGM Whiteness HP group (*p* < 0.05). **Conclusions:** High-concentration (35–40%) in-office bleaching agents demonstrated effective short-term whitening and maintained clinically acceptable and stable color outcomes over 18 months. Although material-dependent differences were observed, bleaching efficacy and long-term color stability appear to be influenced by multiple factors beyond hydrogen peroxide concentration alone. These findings support the clinical reliability of in-office bleaching procedures and highlight the importance of long-term follow-up.

## 1. Introduction

With the growing emphasis on aesthetic dentistry, tooth whitening procedures have gained increasing importance in clinical practice. Tooth discoloration is a common condition that may lead not only to aesthetic concerns but also to psychological and social distress [1]. Various whitening modalities are available for managing discoloration, including in-office procedures, at-home systems and over-the-counter (OTC) products not supervised by dental professionals. Although numerous studies have demonstrated the effectiveness and safety of these approaches, the long-term stability of whitening outcomes remains a subject of ongoing interest, particularly considering variations in lifestyle, dietary habits, and oral hygiene practices [2,3].

Professional whitening procedures have been reported to be influenced by exposure to staining agents such as tea, coffee, and cola; however, recent studies suggest that the extent of this effect may vary depending on treatment protocols and individual factors [4,5,6]. The increased susceptibility of bleached teeth to extrinsic staining has been attributed to structural and chemical alterations of the enamel surface, including increased roughness, permeability, and transient mineral changes. Such alterations may facilitate biofilm and pigment adhesion [7,8]. Therefore, periodic follow-up examinations are essential to monitor color stability and evaluate the long-term success of whitening treatments.

Objective assessment of tooth color is crucial in aesthetic dentistry. Spectrophotometers enable reproducible and highly accurate color measurements by performing spectral analysis within the 400–700 nm wavelength range. They provide color parameters based on the CIE L*, a*, b* system, allowing standardized and digitally compatible analysis. Compared with visual shade selection—which is influenced by operator experience, lighting conditions, and eye fatigue—spectrophotometric measurements offer superior reliability and reproducibility [9,10,11,12].

In-office bleaching is one of the oldest whitening techniques, with a history spanning more than a century. After proper isolation, high-concentration hydrogen peroxide (typically 35–40%) is applied to the tooth surfaces for 20–60 min [13,14]. Hydrogen peroxide remains the most commonly used active ingredient in whitening agents, with concentrations ranging from 15% to 40% depending on the clinical protocol and manufacturer recommendations [15,16,17,18,19].

Hydrogen peroxide-based bleaching systems are widely used and their short-term efficacy has been well documented. However, limited clinical studies have objectively evaluated their long-term effectiveness and color stability through extended patient follow-up, particularly in comparative designs assessing different hydrogen peroxide concentrations.

Therefore, the aim of the present retrospective study was to evaluate the long-term effects of in-office bleaching agents with different hydrogen peroxide concentrations on tooth color change and color stability over a follow-up period of 18 months.

Accordingly, the null hypotheses tested in this study were;

**H1.**  *There is no statistically significant difference in whitening efficacy among the different bleaching agents at the 1-week evaluation following in-office bleaching treatment using high-concentration hydrogen peroxide.*

**H2.**  *There is no statistically significant difference in color change among the different bleaching agents at the 18-month follow-up after in-office bleaching treatment using high-concentration hydrogen peroxide.*

**H3.**  *For each bleaching agent, there is no statistically significant change in color over time.*

## 2. Materials and Methods

The retrospective longitudinal observational study was approved by the Ege University Ethical Committee (approval number: 25-2.1T/76) and conducted in accordance with the Declaration of Helsinki. Written informed consent was obtained from all participants prior to data collection. Data were compiled from patients treated at the Department of Restorative Dentistry at the Ege University. In this retrospective study, the patient record files of 50 patients were used. The patients were treated for bleaching and visited the clinical practice for check-up visits 1 week, 6 months, 12 months and 18 months after the end of the treatment.

The following inclusion criteria were applied:

- Patients (>18 years) with good general oral health who were caries-free without restorations at the labial surfaces and treated in the mentioned unit between 1 November 2022–1 November 2024. The use of 5 different bleaching agents:

G1: Opalesence Boost, Ultradent, South Jordan, UT, USA (40% hydrogen peroxide gel);

G2: Whiteness HP Blue, FGM, Joinville, Brasil (35% hydrogen peroxide gel);

G3: Whiteness, FGM, Joinville, Brasil (35% hydrogen peroxide gel);

G4: Biowhiten, Biodent Ltd., Istanbul, Turkey (40% hydrogen peroxide gel);

G5: Powerbright, Bonegraft, Manisa, Turkey (35% hydrogen peroxide gel)

- The availability of spectrophotometric color values and standardized intraoral photographs at all the study time points (before treatment, immediately after treatment, 1 week, 6 months, 1 year and 18 months after the end of treatment).

Patients with incomplete anamnesis or treatment records, those presenting caries or restorations on the labial surfaces of their teeth, and those who had incomplete records or missing follow-up data were excluded from the study.

**Figure 1:**
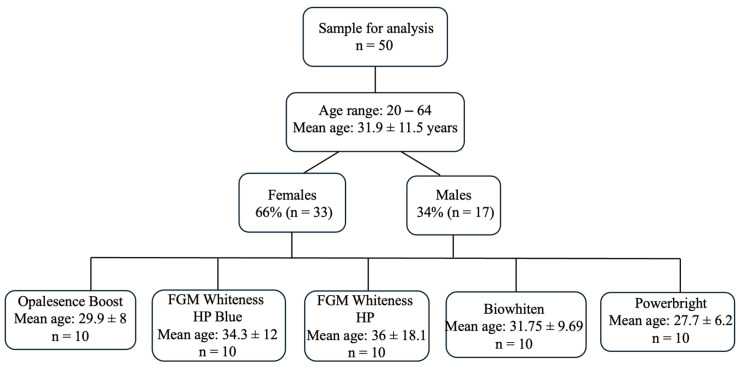
Flow diagram of patient selection and demographics according to STROBE guidelines.

This study was prepared according to the strobe guideline (Supplementary Table S1). Patient records were screened, and individuals who met the inclusion criteria and had complete clinical and follow-up data were included in the study. From this eligible dataset, patients were randomly selected and equally allocated into five bleaching protocol groups (*n* = 10 per group) (Figure 1).

Sample size estimation was performed using G Power 3.1 software with a significance level of 5% and a statistical power of 90%, based on data from a previous study [20]. Considering an anticipated dropout rate of 25%, the final required sample size was calculated as 50 patients in total. Accordingly, 50 patients (278 teeth) who had received bleaching treatment with different hydrogen peroxide-containing bleaching agents were randomly selected from the eligible patient records.

The spectrophotometric evaluation and photographs were performed under standardized conditions. Color measurements were primarily obtained using a spectrophotometer directly from patients during clinical follow-up visits. In addition, standardized clinical photographs available in patient records were also utilized as supportive data for ΔSGU (shade difference) evaluation in the assessment of color changes. An intraoral spectrophotometer (VITA Easyshade V, VITA Zahnfabrik, Bad Säckingen, Germany) was used for the color assessment during the different evaluation times (baseline, after bleaching, 1 week, 6 months, 1 year, 18 months) based on the CIEDE00 system with its formulation:

ΔE_00_ = [(ΔL′/k_L_S_L_)^2^ + (ΔC′/k_C_S_C_)^2^ + (ΔH/k_H_S_H_)^2^ + R_T_ × (ΔC′/k_C_S_C_) × (ΔH′/k_H_S_H_)]^1/2^

To ensure standardization during color assessment, measurements were obtained from the middle third of the buccal surface of the evaluated tooth using a spectrophotometer. A transparent custom-made guide with a 6 mm diameter window, compatible with the tip of the spectrophotometer, was used to standardize the measurement area [21]. All measurements were performed by the same operator under standardized lighting conditions, and the device was calibrated according to the manufacturer’s instructions before each session.

Patient-related behavioral data were collected from clinical records, including smoking status, frequency of consumption of staining beverages (e.g., tea, coffee, and cola), oral hygiene practices (tooth brushing frequency), and use of whitening toothpaste. These variables were included in the analysis to evaluate their potential influence on bleaching outcomes.

The bleaching agents recorded and their composition information obtained from the scanned patient records are presented in Table 1.

Upon retrospective review of the clinical records related to in-office bleaching procedures, it was determined that prior to treatment, the teeth and surrounding soft tissues had been isolated using a light-cured gingival barrier material. Following isolation, the application time and number of sessions varied depending on the bleaching agent used and the manufacturer’s instructions. According to the clinical records, the Opalescence bleaching agent was applied in two 20 min sessions, resulting in a total application time of 40 min. The FGM Whiteness HP Blue product was applied in a single session for a total duration of 40 min. FGM Whiteness HP and Biowhiten products were each applied in three 15 min sessions, totaling 45 min of application time. The Powerbright bleaching agent was applied in a single session with a total duration of 40 min. All in-office bleaching procedures evaluated in this study were performed in accordance with the manufacturers’ instructions as part of routine clinical practice.

Statistical Analysis:

Statistical analysis of ΔE_00_ (color change) and ΔSGU (shade difference) data was performed using SPSS software (version 13.0 for Windows, IBM Corp., Armonk, NY, USA). The normality of data distribution was assessed using the Shapiro–Wilk test. 

Cases with missing data were excluded during the data selection process; therefore, no missing data handling procedures were required in the analysis.

For normally distributed data, one-way repeated measures ANOVA was applied to evaluate the color change of each bleaching agent over time. The assumption of sphericity was tested using Mauchly’s test, and when violated, the Greenhouse–Geisser correction was applied. When a significant difference was detected, pairwise comparisons were performed using the least significant difference (LSD) post hoc test.

For ΔSGU data that did not follow a normal distribution, the Kruskal–Wallis test was used, followed by pairwise comparisons using appropriate non-parametric tests.

To compare differences between bleaching agents, one-way ANOVA was used for ΔE_00_ and the Kruskal–Wallis test was used for ΔSGU at each time point. A significance level of *p* < 0.05 was accepted for all analyses.

Effect sizes were calculated using eta squared (η^2^) to quantify the magnitude of differences between groups and over time. Eta squared values were derived from the ANOVA results (η^2^ = SS_between/SS_total).

Categorical variables, including smoking status, consumption of colored beverages, tooth brushing frequency, and use of whitening toothpaste, were compared among groups using Fisher’s exact test due to the small sample size.

## 3. Results

The present study aimed to evaluate the color change (∆E_00_) and shade difference (ΔSGU) associated with five different in-office bleaching agents (Opalescence, FGM Whiteness HP Blue, FGM Whiteness HP, Biowhiten, and Powerbright) at different time points in a clinical setting at the Department of Restorative Dentistry, Faculty of Dentistry, Ege University. Measurements were taken before treatment, immediately after treatment, at 1 week, 6 months, 1 year, and 18 months. In addition, intergroup comparisons among the bleaching agents were performed.

In the present study, the mean age of the participants was 33.1 ± 11.8 years (range: 20–64), with a relatively homogeneous age distribution across groups, which may have limited the ability to detect the potential influence of age on bleaching outcomes. In addition, baseline color parameters (L*, C*, and h*) were comparable among the groups (L*: 73.5–79.39, C*: 21.12–24.58, h*: 89.21–91.17), with no statistically significant differences observed (*p* > 0.05). In the present study, patient-related behavioral factors were also recorded and analyzed. No statistically significant differences were observed among the groups regarding patients’ habits, including colored beverage consumption (*p* = 0.47), smoking (*p* = 0.73), tooth brushing frequency (*p* = 0.16), and whitening toothpaste use (*p* = 0.81).

Using the ΔE_00_ formula, the color differences between baseline and post-treatment (ΔE_00_1), baseline and week 1 (ΔE_00_2), baseline and 6 months (ΔE_00_3), baseline and 1 year (ΔE_00_4), and baseline and 18 months (ΔE_00_5) were calculated for each patient. Detailed changes in individual color parameters are presented in Appendix A (Table A1, Table A2 and Table A3).

**Figure 2:**
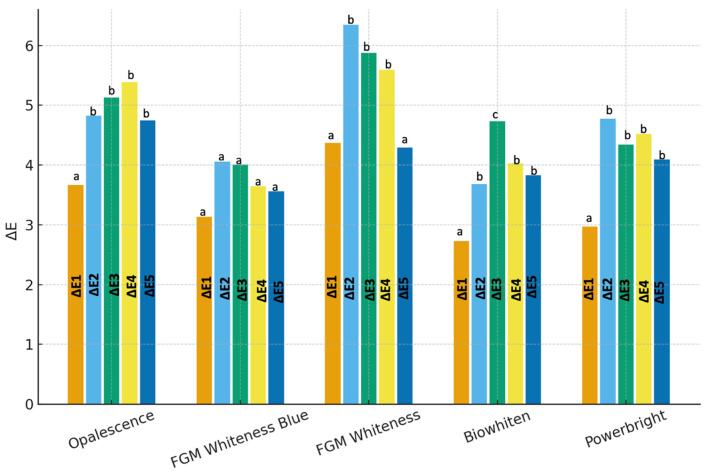
Changes in ΔE_00_ values over time for the evaluated in-office bleaching agents (Opalescence, FGM Whiteness HP Blue, FGM Whiteness HP, Biowhiten, and Powerbright). Different lowercase letters (a–c) above the bars indicate statistically significant differences among time points within each group (*p* < 0.05). Values are presented as mean ΔE_00_.

Intragroup Evaluation of Color Change (ΔE_00_):

Statistically significant changes in ΔE_00_ values over time were observed in the Opalescence group (*p* < 0.05). The ΔE_00_1 value was significantly lower than those recorded at all subsequent time points (ΔE_00_2–ΔE_00_5), whereas no statistically significant differences were detected among ΔE_00_2, ΔE_00_3, ΔE_00_4, and ΔE_00_5. These findings indicate that the whitening effect was achieved rapidly after treatment and remained stable throughout the follow-up period (Figure 2/Table 2).

In the FGM Whiteness HP Blue group, no statistically significant differences were observed in ΔE_00_ values across time points (*p* > 0.05), indicating that the whitening effect remained stable over time without significant color variation during the follow-up period (Figure 2/Table 2).

In contrast, in the FGM Whiteness HP group, repeated-measures ANOVA revealed statistically significant differences among time points (*p* < 0.05). The ΔE_00_1 value was significantly lower than ΔE_00_2, ΔE_00_3, and ΔE_00_4, while no statistically significant difference was found between ΔE_00_1 and ΔE_00_5. In addition, ΔE_00_5 values were significantly lower than ΔE_00_2, ΔE_00_3, and ΔE_00_4, whereas no significant differences were detected among ΔE_00_2, ΔE_00_3, and ΔE_00_4. These findings suggest that a pronounced whitening effect was achieved following treatment, which remained stable up to the 12-month follow-up, with a partial relapse observed at the 18-month evaluation (Figure 2/Table 2).

In the Biowhiten group, the ΔE_00_1 value was significantly lower than all other time points, whereas ΔE_00_3 exhibited the highest values and was significantly higher than all other measurements. No statistically significant differences were observed among ΔE_00_2, ΔE_00_4, and ΔE_00_5 (*p* > 0.05). These findings indicate that although a substantial whitening effect was achieved, a degree of color regression occurred over time (Figure 1/Table 2).

In the Powerbright group, statistically significant differences in ΔE_00_ values were observed over time (*p* < 0.05). The ΔE_00_1 value was significantly lower than all subsequent time points, while no statistically significant differences were found among ΔE_00_2, ΔE_00_3, ΔE_00_4, and ΔE_00_5. These results demonstrate that the whitening effect occurred rapidly after treatment and remained stable throughout the follow-up period (Figure 1/Table 2).

All bleaching agents exceeded both perceptibility (PT = 1.2) and acceptability (AT = 2.7) thresholds at 1 week. ΔE_00_ values remained above the perceptibility threshold at all time points, while acceptability was generally maintained over 18 months, with a slight decrease observed in the FGM Whiteness HP group (Figure 1/Table 2).

**Figure 3:**
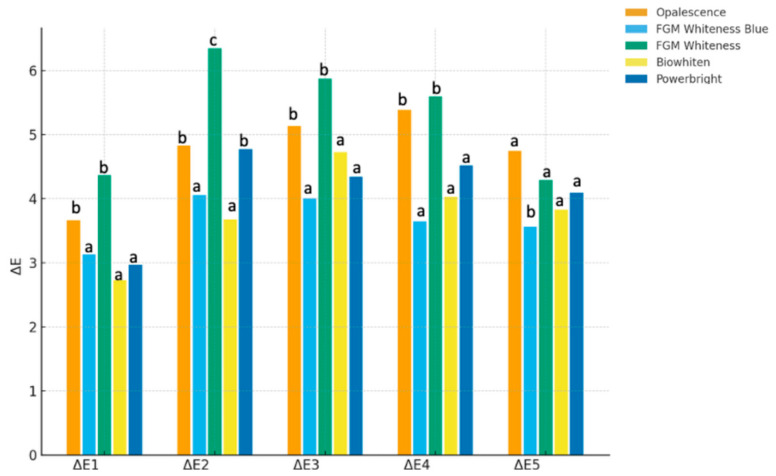
Intergroup comparison of ΔE_00_ values of the evaluated in-office bleaching agents at different time points (ΔE_00_1–ΔE_00_5). Different lowercase letters (a–c) above the bars indicate statistically significant differences among groups within the same time point (*p* < 0.05). Values are presented as mean ΔE_00_.

Intergroup Comparison of Color Change (ΔE_00_):

Intergroup comparisons revealed that the bleaching agents exhibited different ΔE_00_ profiles across time points (Figure 3/Table 2).

At ΔE_00_1, FGM Whiteness HP and Opalescence showed similarly high ΔE_00_ values, which were significantly greater than those of Biowhiten, Powerbright, and FGM Whiteness HP Blue (*p* < 0.05).

At ΔE_00_2 and ΔE_00_3, FGM Whiteness HP demonstrated the highest values, significantly exceeding all other groups (*p* < 0.05), while Opalescence showed intermediate values and the remaining agents exhibited lower and comparable results.

At ΔE_00_4, FGM Whiteness HP and Opalescence presented similarly high values, both significantly greater than those of the other groups (*p* < 0.05).

At ΔE_00_5, Opalescence exhibited the highest ΔE_00_ values; however, these were not statistically different from FGM Whiteness HP, Powerbright, or Biowhiten, whereas FGM Whiteness HP Blue showed significantly lower values compared with the other agents (*p* < 0.05).

Statistically significant differences were observed between bleaching agents across multiple time points (*p* < 0.05). In addition to statistical significance, effect sizes were calculated to assess the magnitude of differences. The analysis revealed moderate to large effect sizes for the differences between bleaching agents (η^2^ ranging from 0.08 to 0.16). Similarly, the effect of time on ΔE_00_ values demonstrated moderate to large effect sizes, with the highest observed at ΔE_2_ (η^2^ ≈ 0.20). These findings indicate that the observed differences are not only statistically significant but also clinically meaningful.

Shade Difference (ΔSGU):

The ΔSGU_1_ values, representing the baseline-end-of-session interval, were statistically significantly lower than those of all subsequent time points (*p* < 0.05). No statistically significant differences were observed among ΔSGU_2_, ΔSGU_3_, ΔSGU_4_, and ΔSGU_5_ within each group.

**Figure 4:**
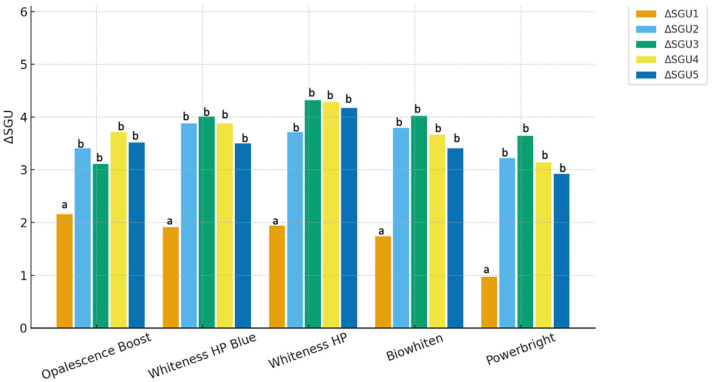
Intergroup Comparison of Changes in Whitening Efficacy (ΔSGU) Over Time. Different lowercase letters (a,b) above the bars indicate statistically significant differences among groups within the same time point (*p* < 0.05).

Intergroup comparisons at each time point revealed generally comparable ΔSGU values among the evaluated bleaching agents, with no consistent statistically significant differences observed between groups (*p* > 0.05) (Figure 4/Table 3).

The time-dependent changes in mean ΔSGU values for each agent are presented in Figure 3. These findings indicate that all bleaching agents produced a similar pattern of shade change over time, with no marked differences in clinical performance based on ΔSGU measurements.

The combined evaluation of ΔSGU and ΔE_00_ values revealed statistically significant differences among the bleaching agents in terms of ΔE_00_ at certain measurement time points (*p* < 0.05). In contrast, ΔSGU values were generally comparable among the agents, with no consistent statistically significant differences observed across the evaluated time points (*p* > 0.05).

## 4. Discussion

Esthetic dental appearance, particularly tooth color, plays an important role in patients’ self-esteem and overall well-being. The increasing demand for conservative and minimally invasive esthetic treatments has contributed to the widespread use of tooth bleaching procedures [22]. Among available techniques, professionally supervised in-office bleaching remains a preferred option due to its predictable results and controlled clinical application [3,23]. Hydrogen peroxide-based agents achieve whitening through oxidative degradation of chromogenic molecules; however, associated changes in enamel permeability may influence post-treatment color stability [24,25,26]. Therefore, evaluating both short- and long-term outcomes of in-office bleaching protocols is clinically relevant [27].

Recent bibliometric evidence has demonstrated a clear shift in bleaching research trends, moving beyond the evaluation of immediate whitening efficacy toward a broader focus on long-term color stability, treatment protocols, and associated side effects such as tooth sensitivity. In this context, Aragão et al. (2025) highlighted that although in-office bleaching with 35% hydrogen peroxide remains the most extensively investigated approach, there is still a significant lack of long-term clinical data assessing the durability of whitening outcomes under real clinical conditions [28].

The present study directly addresses this gap by providing 18-month follow-up data derived from a clinical setting, thereby contributing to the limited body of evidence on long-term bleaching stability. Furthermore, consistent with recent literature trends, the findings of this study suggest that bleaching efficacy and color stability cannot be explained solely by hydrogen peroxide concentration, but are likely influenced by a combination of formulation characteristics and clinical variables. Accordingly, in this study, clinical records of patients who underwent in-office bleaching treatment at the Department of Restorative Dentistry, Ege University, between 1 November 2022–1 November 2024 were retrospectively evaluated. The whitening efficacy of different bleaching agents and their long-term color stability were assessed.

Accurate assessment of tooth color is essential for evaluating bleaching outcomes. Visual shade matching is influenced by multiple variables, including lighting conditions, observer experience, eye fatigue, measurement angle, and environmental factors. To minimize subjectivity and enhance reproducibility, digital spectrophotometers have been widely adopted [29,30]. These devices provide standardized CIE L*, a*, and b* values and can detect differences beyond human visual perception. The Vita EasyShade spectrophotometer, validated for reliability and repeatability in previous studies [31,32,33], was therefore selected for this study.

In this study color differences between baseline and follow-up sessions were calculated using the CIEDE2000 (ΔE_00_) formula, which incorporates perceptual corrections for lightness, chroma, and hue. Compared with the conventional CIELab formula, ΔE_00_ more accurately reflects human visual perception in dental applications [34,35]. In addition to objective spectrophotometric evaluation, visual shade guide units (SGU) were also assessed. Although ΔSGU values varied significantly over time within groups, no statistically significant differences were detected between bleaching agents. This finding suggests that instrumental measurements may detect subtle color differences that are not necessarily perceptible under clinical conditions. Therefore, relying solely on objective parameters may not fully reflect clinical success, and perceptibility thresholds should be considered when interpreting bleaching outcomes. In this context, the integration of objective spectrophotometric data with visual assessment methods may provide a more comprehensive and clinically relevant evaluation of whitening efficacy.

According to the CIEDE2000 system, a post-bleaching color difference (ΔE_00_) exceeding 3.3, together with an approximate four-shade improvement on the Vita Classical shade guide, indicates clinically successful treatment [36]. In the present study, the ΔE_00_ values obtained at the 1-week follow-up—considered the time point at which post-bleaching color stabilization occurs—were above the 3.3 threshold in all agent groups. This finding demonstrates that the color changes achieved following treatment were clinically perceptible to the naked eye. Although lower ΔE_00_ values were observed in the Biowhiten and Powerbright Plus groups compared with the other agents, these values remained within clinically acceptable limits, indicating that these agents may also be considered effective and reliable bleaching materials.

It has been reported that a stabilization period is required for the accurate assessment of color change following bleaching treatment. The literature emphasizes that a minimum follow-up period of one week is necessary to allow post-bleaching color stabilization to occur [32,37]. In the present study, analysis of the data revealed that, in all agent groups except FGM Whiteness HP Blue, the ΔE_001_ values obtained immediately after treatment were significantly lower than the ΔE_002_ values representing the stabilization period. This finding suggests that the color changes observed immediately after bleaching differ from those measured following stabilization, possibly due to the continued action of residual oxygen radicals on the tooth surface after completion of the bleaching procedure. Therefore, in the present study, the ΔE_002_ values—considered to reflect stabilized color—were used as the reference parameter for evaluating the true clinical effectiveness of the bleaching treatment. This observation also suggests that early post-treatment evaluations may underestimate the actual whitening outcome, highlighting the importance of appropriate timing in clinical color assessment.

Baseline determinants such as initial tooth color, age, and dental or medical history are well-established modulators of bleaching efficacy. In particular, darker baseline shades have been associated with greater perceptible color change, whereas age-related alterations in enamel and dentin may influence peroxide diffusion and treatment responsiveness. In the present study, age distribution and baseline color parameters were comparable across all groups, indicating a high degree of initial homogeneity. In addition, patient-related behavioral factors, including smoking and the consumption of chromogenic beverages such as tea, coffee, and cola, did not differ significantly among groups. This methodological consistency minimizes the potential influence of confounding variables and strengthens the internal validity of the findings, suggesting that the observed differences in bleaching outcomes are primarily attributable to treatment-related factors rather than baseline or behavioral variability. Nevertheless, given the relatively limited sample size, the potential contribution of these variables cannot be entirely excluded and should be interpreted with caution. It should also be noted that, although baseline tooth color was assessed, other biological determinants—such as enamel ultrastructure, saliva composition, and individual mineral content—were beyond the scope of this study, representing an inherent limitation [27,32,37].

Within this context, bleaching outcomes should be interpreted as the result of a multifactorial process rather than being solely dependent on hydrogen peroxide concentration. Contemporary evidence increasingly emphasizes the combined influence of formulation characteristics, clinical protocols, and patient-related factors in determining both immediate whitening efficacy and long-term color stability. In agreement with this perspective, Aragão et al. (2025) highlighted a shift in the literature toward a more comprehensive evaluation of bleaching systems, underscoring the importance of considering multiple interacting variables when assessing treatment success [28].

Accordingly, the role of hydrogen peroxide concentration alone appears insufficient to fully explain differences in bleaching performance. Although high-concentration agents are known to produce rapid and clinically perceptible whitening effects, previous studies have demonstrated that increasing peroxide concentration does not necessarily translate into greater color change [20]. For instance, Altınışık et al. (2023) reported no significant enhancement in whitening efficacy with higher peroxide concentrations, while Lee et al. (2019) showed that a 25% hydrogen peroxide system combined with light activation could outperform a 40% system, highlighting the critical role of formulation and application protocols [24,38]. Furthermore, high-concentration bleaching agents have been associated with increased surface roughness, which may predispose teeth to color relapse over time. In the present study, all bleaching agents contained relatively high hydrogen peroxide concentrations (35–40%), precluding a direct comparison based solely on concentration. Therefore, the observed differences in whitening efficacy are more plausibly attributed to variations in formulation characteristics and application protocols, reinforcing the concept that bleaching performance is governed by a complex interplay of material- and protocol-dependent factors.

Nevertheless, treatment success should not be evaluated solely based on short-term color change. One of the most common concerns expressed by patients is the persistence of the whitening effect over time. The phenomenon referred to as the “rebound effect” has been associated with renewed pigment accumulation and structural changes in the enamel, such as increased porosity or mineral loss, resulting from biological and environmental factors [32,39].

Previous studies have consistently reported that in-office bleaching procedures achieve high short-term efficacy, with the greatest color change occurring within the first days or months following treatment [32,33,40]. While color stability is generally maintained during the first year, several studies have documented a gradual color relapse of approximately 10% over longer follow-up periods [32,40]. Methodological heterogeneity among studies, including differences in colorimetric indices, follow-up durations, and bleaching protocols, complicates direct comparisons of long-term outcomes [29,39].

The 18-month color changes observed in the present study were generally consistent with previously reported long-term bleaching outcomes. In this study, which evaluated in-office protocols using high-concentration hydrogen peroxide (HP), no marked color relapse was observed during the first 6–12 months, except for minor fluctuations in the Biowhiten group. However, a rebound tendency was observed at the 18-month follow-up, which reached statistical significance only in the FGM Whiteness HP group, while remaining non-significant in the other groups. Overall, this relapse was clinically limited despite its statistical significance in specific cases.

In the present study, the FGM Whiteness HP group exhibited a significant color regression at the 18-month evaluation, indicating reduced long-term color stability compared with other bleaching agents. This finding suggests that formulation-related factors, particularly the relatively lower pH of this agent (approximately 5.7), may play an important role in its long-term performance. Although the bleaching agents contained similar hydrogen peroxide concentrations, their clinical performance differed, indicating that peroxide concentration alone does not determine whitening efficacy. The pH of bleaching agents plays a critical role in the chemical behavior of hydrogen peroxide. Acidic environments may accelerate the decomposition of hydrogen peroxide into reactive oxygen species, potentially enhancing initial bleaching efficacy. However, lower pH values may also induce greater alterations in enamel microstructure, including increased surface roughness and porosity. These changes may facilitate the penetration and retention of chromogenic agents, thereby increasing susceptibility to extrinsic staining and contributing to long-term color relapse. In contrast, agents with neutral or slightly alkaline pH may provide more controlled peroxide activity and improved long-term color stability [24,41].

The lack of significant change over time observed in the FGM Whiteness HP Blue group may be attributed to its comparatively lower ΔE_00_ values and milder bleaching effect, which could limit the extent of detectable color variation during follow-up. Additionally, formulation-related factors—such as its relatively neutral to slightly alkaline pH and the presence of stabilizing components (e.g., calcium-containing compounds)—may promote a more controlled release of reactive oxygen species, contributing to a more stable and uniform color profile over time.

From a clinical perspective, these findings suggest that the selection of bleaching agents should not be based solely on hydrogen peroxide concentration. Instead, formulation characteristics such as pH should be carefully considered, as they may influence both the immediate whitening effect and long-term color stability. These findings are consistent with previous studies indicating that the efficacy of hydrogen peroxide-based bleaching systems is strongly influenced by pH-dependent oxidative dynamics, where acidic conditions may promote rapid but less stable effects, whereas neutral or alkaline conditions may support more sustained whitening outcomes [42,43].

To evaluate agent-specific performance, ΔE_00_ values obtained at the 1-week stabilization period (ΔE_00_2) were compared with those at the 18-month follow-up (ΔE_00_5). No statistically significant differences were found between these time points for any agent except FGM Whiteness HP. However, although a statistically significant reduction was observed in this group, the ΔE_005_ value remained above the clinical perceptibility threshold, indicating that the whitening effect was still clinically meaningful over the long term.

Overall, these findings suggest that the evaluated bleaching agents generally maintained their whitening efficacy over time, despite minor or agent-specific variations in color stability.

This finding is particularly relevant in light of recent bibliometric analyses indicating a lack of extended follow-up clinical studies in the bleaching literature. In this regard, the present study contributes to addressing this gap by providing 18-month clinical data under real-world conditions.

When the findings of the present study are considered together with the existing literature, it can be inferred that initial whitening outcomes may vary depending on the bleaching agent used, whereas long-term color stability appears to be influenced by a complex interaction of material-related, biological, and patient-dependent factors [44,45].

Comparative evaluation of the in-office bleaching agents used in this study revealed statistically significant differences in bleaching efficacy at several evaluation time points. Among the tested products, FGM Whiteness HP generally demonstrated higher ΔE_00_ values, indicating greater whitening efficacy, followed by the Opalescence group. In contrast, Biowhiten, Powerbright, and FGM Whiteness HP Blue exhibited comparatively lower ΔE_00_ values.

Biowhiten differs from the other agents by containing nano-hydroxyapatite (nHA). Although nHA has been reported to promote remineralization and reduce enamel porosity, it may also limit the diffusion of hydrogen peroxide into deeper enamel layers [46,47,48]. This may partially explain the relatively lower ΔE_00_ values observed in this group. However, the similarly lower whitening efficacy observed in the FGM Whiteness HP Blue group, which does not contain nHA, suggests that reduced bleaching performance cannot be attributed solely to this component and is likely influenced by multiple formulation-related factors.

In light of these findings, the high-concentration in-office bleaching agents evaluated in this study demonstrated strong short-term efficacy, and the color change achieved during the first year was largely maintained. Although limited changes in ΔE_00_ values were observed at the 18-month follow-up, these did not represent a consistent overall trend.

Regarding whitening efficacy at the stabilization period (ΔE_00_2), statistically significant differences were identified among the in-office bleaching agents (*p* < 0.05). Despite comparable hydrogen peroxide concentrations, the observed variations in clinical outcomes indicate that whitening efficacy cannot be attributed solely to peroxide concentration. Factors such as application time, activation protocol, gel formulation, stabilizer content, and baseline tooth color may also contribute to treatment effectiveness. Accordingly, the null hypothesis (H1) was rejected.

Evaluation of long-term outcomes at 18 months revealed statistically significant differences in color change among the bleaching agents (*p* < 0.05). Post hoc analysis showed that the ΔE_00_5 values of the FGM Whiteness HP Blue group were significantly lower than those of the other agents, whereas no statistically significant differences were observed among Opalescence, FGM Whiteness HP, Biowhiten, and Powerbright.

The lack of a statistically significant association between patient-related habits and long-term color change suggests that variations in long-term outcomes may be more strongly related to material- and application-dependent factors rather than individual behavioral variables. Accordingly, the null hypothesis (H2) was rejected.

When time-dependent color changes were analyzed, whitening efficacy was found to vary over time for most of the evaluated agents. While pronounced color improvement was observed in the early post-treatment period, subsequent evaluations demonstrated either stabilization or partial regression depending on the agent. These findings indicate that bleaching treatment is not a static process but rather a dynamic phenomenon influenced by chemical and microstructural alterations of the enamel surface, as well as individual biological factors. Long-term clinical performance may be affected by enamel microstructural changes, saliva-induced remineralization processes, and exposure to environmental staining agents. Since the bleaching agents did not demonstrate consistent stability over time, the null hypothesis (H3) was rejected.

## 5. Conclusions

Within the limitations of this retrospective clinical study, high-concentration (35–40%) in-office bleaching agents demonstrated clinically effective short-term whitening, with ΔE_00_ values exceeding perceptibility thresholds in all groups at the stabilization period. Among the evaluated systems, FGM Whiteness HP and Opalescence exhibited higher whitening efficacy, whereas Biowhiten, Powerbright, and FGM Whiteness HP Blue showed comparatively lower, yet clinically acceptable, outcomes.

Despite these differences in initial efficacy, all bleaching agents generally maintained stable color outcomes over the 18-month follow-up period. Importantly, no clinically meaningful long-term color deterioration was observed, although minor and agent-specific rebound effects were detected, reaching statistical significance only in the FGM Whiteness HP group.

The findings of the present study further support that bleaching efficacy cannot be explained by hydrogen peroxide concentration alone. Instead, the observed variations in both short-term performance and long-term stability suggest a relevant influence of formulation-related factors, particularly those associated with application protocols and material characteristics. In this context, the significant color regression observed in the FGM Whiteness HP group may be associated with its lower pH compared with other evaluated systems, indicating that formulation parameters may play a role in long-term color stability.

Importantly, these findings are consistent with recent developments in bleaching research, where increasing attention has been directed toward the evaluation of long-term outcomes rather than immediate whitening efficacy alone. Within this perspective, the present study contributes to the existing literature by providing extended clinical follow-up data and by highlighting the importance of formulation-dependent differences in bleaching performance.

From a clinical standpoint, in-office bleaching remains a reliable and effective esthetic treatment modality. However, the selection of bleaching agents should not be based solely on initial whitening efficacy, but should also consider long-term color stability and material characteristics. The combined use of objective spectrophotometric measurements and clinical evaluation is therefore essential for a comprehensive assessment of treatment success.

Nevertheless, the retrospective design, potential selection bias, and reliance on self-reported behavioral data represent important limitations of the present study. The retrospective nature of the study limits control over confounding variables, while selection bias may have influenced the composition of the study population. In addition, reliance on self-reported behavioral factors, such as dietary habits, oral hygiene practices, and smoking status, may introduce reporting bias and variability, potentially affecting the accuracy of the observed color stability outcomes. Furthermore, the findings may have limited generalizability to broader populations with different characteristics. Therefore, future prospective, randomized, and standardized clinical studies with extended follow-up periods are required to better elucidate the factors influencing bleaching efficacy and long-term color stability.

## Figures and Tables

**Table 1:** Bleaching agents and their compositions.

Commercial Product	Active Ingredient	Composition	pH	Total Exposure Time to Agent	Preparation	Application
Opalescence Boost, Ultradent, South Jordan, UT, USA	%40 HP	40% hydrogen peroxide, potassium nitrate, potassium hydroxide, sodium fluoride, dimethicone, glycerin	7	40 min	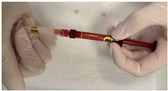	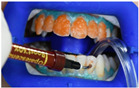
Whiteness HP Blue, FGM, Joinville, Brasil	%35 HP	35% hydrogen peroxide, thickening agents, violet pigment, neutralizing agents, calcium gluconate, glycol and water	8–9	40 min	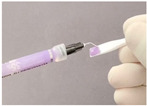	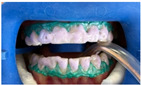
Whiteness HP, FGM, Joinville, Brasil	%35 HP	35% hydrogen peroxide, thickening agents, violet pigment, neutralizing agents, glycol and water	5.7	45 min	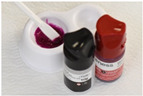	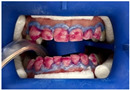
Biowhiten, Biodent Ltd., Istanbul, Turkey	%40 HP	Water, glycerin, alcohol, sodium bicarbonate, sodium hydroxide, 25% hydrogen peroxide and nHA (nano-hydroxyapatite)	>7.5	45 min	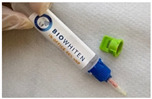	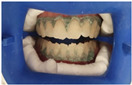
Powerbright Bonegraft, Manisa, Turkey	%35 HP	35% hydrogen peroxide, glycerol, sodium hydroxide, potassium nitrate, carbomer	6.5	40 min	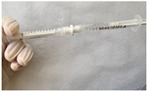	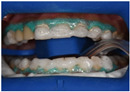

**Table 2:** Distribution of Color Change (ΔE_00_) Results of Groups with Different Agent Compositions.

		ΔE_00_1	ΔE_00_2	ΔE_00_3	ΔE_00_4	ΔE_00_5
Opalescence Boost	Mean	3.66 ^aB^ 0.39	4.82 ^bB^ 0.25	5.13 ^bB^ 0.31	5.38 ^bB^ 0.35	4.74 ^bA^ 0.25
	SD					
FGM Whiteness HP Blue	Mean	3.13 ^aA^ 0.32	4.05 ^aA^ 0.31	4.01 ^aA^ 0.28	3.64 ^aA^ 0.26	3.56 ^aB^ 0.25
	SD					
FGM Whiteness HP	Mean	4.36 ^aB^ 0.68	6.34 ^bC^ 0.31	5.87 ^bB^ 0.32	5.59 ^bB^ 0.37	4.28 ^aA^ 0.35
	SD					
Biowhiten	Mean	2.72 ^aA^ 0.25	3.67 ^bA^ 0.23	4.73 ^cA^ 0.24	4.02 ^bA^ 0.21	3.82 ^bA^ 0.19
	SD					
Powerbright	Mean	2.97 ^aA^ 0.21	4.77 ^bB^ 0.27	4.34 ^bA^ 0.27	4.51 ^bA^ 0.27	4.09 ^bA^ 0.25
	SD					

Values within the same row that bear different lowercase letters indicate statistically significant differences between time points. Values within the same column that share the same uppercase letters indicate no statistically significant differences among agents (*p* < 0.05).

**Table 3:** Distribution of Color Change (ΔSGU) Results of Groups with Different Agent Compositions.

		ΔSGU1	ΔSGU2	ΔSGU3	ΔSGU4	ΔSGU5
Opalescence Boost	Mean	0.97 ^aA^ 0.41	3.41 ^bA^ 0.51	3.11 ^bA^ 0.52	3.14 ^bA^ 0.55	2.92 ^bA^ 0.53
	SD					
FGM Whiteness HP Blue	Mean	1.91 ^aA^ 0.46	3.88 ^bA^ 0.54	4.01 ^bA^ 0.56	3.88 ^bA^ 0.52	3.5 ^bA^ 0.57
	SD					
FGM Whiteness HP	Mean	1.94 ^aA^ 0.54	3.71 ^bA^ 0.59	4.32 ^bA^ 0.52	4.29 ^bA^ 0.56	4.17 ^bA^ 0.56
	SD					
Biowhiten	Mean	1.74 ^aA^ 0.52	3.79 ^bA^ 0.52	4.02 ^bA^ 0.59	3.67 ^bA^ 0.57	3.41 ^bA^ 0.56
	SD					
Powerbright	Mean	2.16 ^aA^ 0.39	3.22 ^bA^ 0.45	3.64 ^bA^ 0.51	3.72 ^bA^ 0.50	3.52 ^bA^ 0.51
	SD					

Values within each row that bear different lowercase letters indicate statistically significant differences between time points. Values within the same column that share the same uppercase letters indicate no statistically significant differences among agents (*p* > 0.05).

## Data Availability

The original contributions presented in the study are included in the article, further inquiries can be directed to the corresponding author.

## Supplementary Materials

The following supporting information can be downloaded at: https://www.mdpi.com/article/10.3390/jcm15093458/s1, Table S1: STROBE checklist for observational studies.

## Appendix A

Changes in individual color parameters (ΔL*, Δa*, and Δb*) are presented in Appendix Table A1, Table A2 and Table A3.

**Table A1:** Changes in lightness (ΔL*) across time points.

Group	T2-T1 (Mean ± SD)	T3-T1 (Mean ± SD)	T4-T1 (Mean ± SD)	T5-T1 (Mean ± SD)	T6-T1 (Mean ± SD)
Opalescence Boost	+1.87 ± 4.37	+3.39 ± 3.61	+1.09 ± 4.02	+0.89 ± 3.36	+1.63 ± 3.22
FGM Whiteness HP Blue	1.63 ± 2.74	1.33 ± 3.36	1.49 ± 3.01	0.62 ± 2.98	0.57 ± 3.18
FGM Whiteness HP	0.76 ± 3.52	1.43 ± 3.96	1.69 ± 3.38	0.90 ± 3.41	0.66 ± 3.12
Biowhiten	1.63 ± 3.07	1.64 ± 4.78	0.55 ± 4.57	1.04 ± 4.49	0.92 ± 4.36
Powerbright	0.41 ± 2.77	−0.63 ± 3.35	0.84 ± 2.95	0.32 ± 3.73	0.67 ± 3.41

Δ values were calculated as the difference between each follow-up measurement and baseline (T1). T2, T3, T4, T5, and T6 represent subsequent evaluation time points.

**Table A2:** Changes in redness–greenness parameter (Δa*) across time points.

Group	T2-T1 (Mean ± SD)	T3-T1 (Mean ± SD)	T4-T1 (Mean ± SD)	T5-T1 (Mean ± SD)	T6-T1 (Mean ± SD)
Opalescence Boost	−0.69 ± 0.96	−1.16 ± 1.02	−1.08 ± 0.99	−1.18 ± 1.01	−1.07 ± 1.00
FGM Whiteness HP Blue	−0.68 ± 0.88	−1.05 ± 0.91	−1.03 ± 0.89	−1.13 ± 0.87	−1.05 ± 0.88
FGM Whiteness HP	−0.66 ± 0.94	−1.09 ± 1.01	−1.04 ± 0.97	−1.16 ± 0.98	−1.05 ± 0.99
Biowhiten	−0.74 ± 0.93	−1.06 ± 0.94	−0.71 ± 0.89	−1.00 ± 0.89	−1.07 ± 0.89
Powerbright	−0.78 ± 0.91	−1.09 ± 0.95	−0.94 ± 0.93	−1.05 ± 0.92	−1.11 ± 0.93

Δ values were calculated as the difference between each follow-up measurement and baseline (T1).

**Table A3:** Changes in yellowness–blueness parameter (Δb*) across time points.

Group	T2-T1 (Mean ± SD)	T3-T1 (Mean ± SD)	T4-T1 (Mean ± SD)	T5-T1 (Mean ± SD)	T6-T1 (Mean ± SD)
Opalescence Boost	−0.73 ± 3.02	−4.35 ± 3.48	−5.27 ± 3.61	−5.94 ± 3.82	−5.62 ± 3.74
FGM Whiteness HP Blue	−0.52 ± 2.83	−4.04 ± 3.26	−5.10 ± 3.41	−5.59 ± 3.56	−5.32 ± 3.48
FGM Whiteness HP	−0.58 ± 3.01	−4.12 ± 3.44	−5.02 ± 3.59	−5.61 ± 3.76	−5.29 ± 3.68
Biowhiten	−0.69 ± 2.74	−3.23 ± 3.02	−4.64 ± 3.52	−5.34 ± 3.73	−6.27 ± 3.81
Powerbright	−0.61 ± 2.96	−4.27 ± 3.42	−5.18 ± 3.61	−5.86 ± 3.84	−5.48 ± 3.72

Δ values were calculated as the difference between each follow-up measurement and baseline (T1). T2, T3, T4, T5, and T6 represent subsequent evaluation time points.
